# Thymoquinone Inhibits *Escherichia coli* ATP Synthase and Cell Growth

**DOI:** 10.1371/journal.pone.0127802

**Published:** 2015-05-21

**Authors:** Zulfiqar Ahmad, Thomas F. Laughlin, Ismail O. Kady

**Affiliations:** 1 Department of Biochemistry, Kirksville College of Osteopathic Medicine, A T Still University of Health Sciences, Kirksville, MO, 63501, United States of America; 2 Department of Chemistry, East Tennessee State University, Johnson City, TN, 37614, United States of America; University of Alabama at Birmingham, UNITED STATES

## Abstract

We examined the thymoquinone induced inhibition of purified F_1_ or membrane bound F_1_F_O_ *E*. *coli* ATP synthase. Both purified F_1_ and membrane bound F_1_F_O_ were completely inhibited by thymoquinone with no residual ATPase activity. The process of inhibition was fully reversible and identical in both membrane bound F_1_F_o_ and purified F_1_ preparations. Moreover, thymoquinone induced inhibition of ATP synthase expressing wild-type *E*. *coli* cell growth and non-inhibition of ATPase gene deleted null control cells demonstrates that ATP synthase is a molecular target for thymoquinone. This also links the beneficial dietary based antimicrobial and anticancer effects of thymoquinone to its inhibitory action on ATP synthase.

## Introduction

ATP synthase is the principal energy generating enzyme in all organisms from bacteria to vertebrates through oxidative phosphorylation or photophosphorylation. This is a highly conserved enzyme with two sectors F_1_ and F_o_. F_1_ is composed of α_3_ß_3_γδε and F_o_ of ab_2_c_10–14_. ATP hydrolysis and synthesis occur on three catalytic sites in the F_1_ sector, whereas proton movement occurs through the membrane embedded F_o_ [1,2]. A transmembrane proton gradient allows the flow of protons through the F_o_ sector. Proton gradient-driven rotation of γ-subunit causes conformational changes in the α/β subunits which in turn results in ATP synthesis or hydrolysis depending on the direction of the proton gradient. This terminal enzyme of oxidative phosphorylation is also the smallest known biological nanomotor [3,4,5,6].

ATP synthase is an important molecular drug target for many diseases, like cancer, tuberculosis, obesity, and microbial infections [7,8,9]. The presence of ectopic ATP synthase in particular can make it an attractive drug target in a number of cellular processes. For example, inhibition of ATP synthase has been suggested as an anti-angiogenic therapeutic to block tumor angiogenesis [10] and a decrease in lung carcinoma was observed by inhibiting ectopic ATP synthase [11]. Blocking the synthesis of ATP by targeting subunit c of ATP synthase is being used to treat tuberculosis [12]. Another drug, Bz-423 that induces apoptosis in lymphoid cells, has been found to inhibit the mitochondrial ATP synthase [13]. Also, it is been shown that ectopic ATP synthase may be a suitable molecular target for inhibiting HIV-1 proliferation in vivo [14].

A variety of natural and modified plant based molecules are known to induce either complete or partial inhibition of ATP synthase with potential resulting health benefits [7,15,16,17,18]. Some health benefits of fruits, vegetables, and other phytochemicals are credited to the polyphenols present in them. These phytochemicals are known for their antioxidants, chemopreventive, chemotherapeutic, and anti-microbial properties [7,19,20,21,22,23]. Some dietary polyphenolic compounds were shown to block the action of cell constituents that promote growth of tumor cells by binding to the multiple molecular targets in the body, including ATP synthase [19,24].

**Fig 1:**
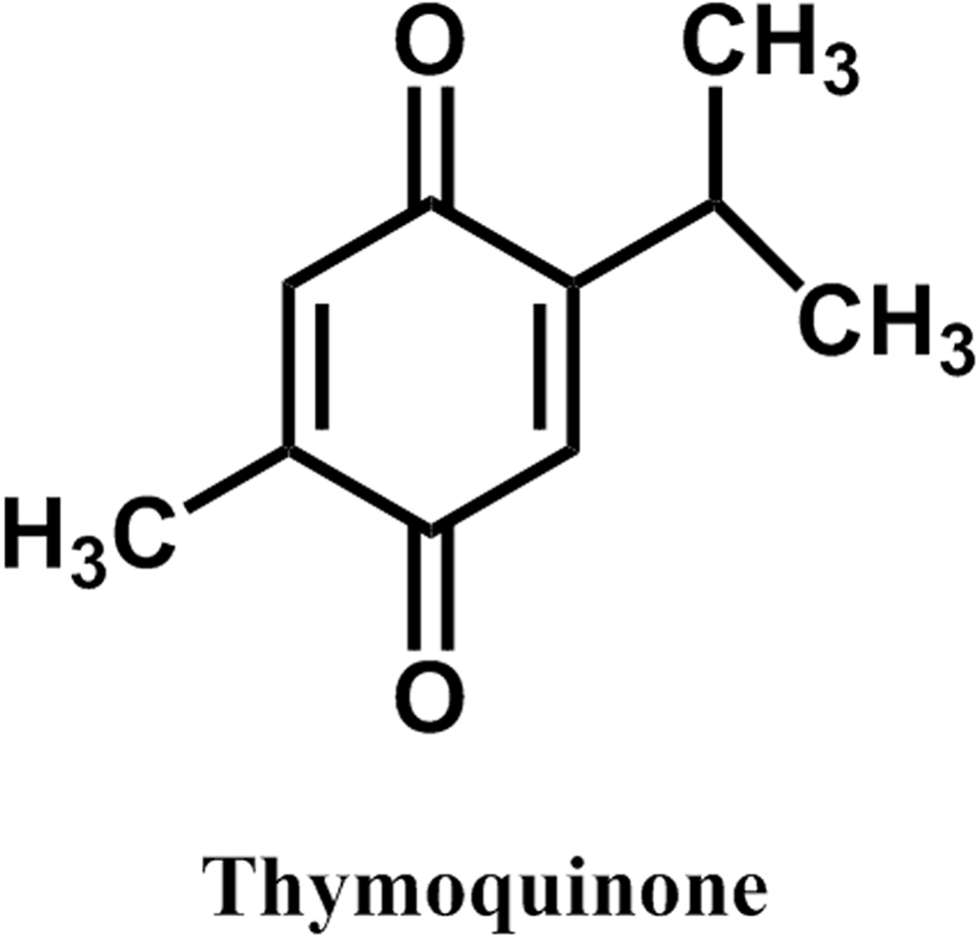
Structures of thymoquinone (TQ).

Thymoquinone (TQ) is a major phytochemical compound found in the medicinal plant *Nigella sativa* an annual flowering plant in the family *Ranunculaceae* (Fig 1). Thymoquinone has been tested against many cancer cell lines and has exhibited potent inhibitory effects on lung, prostate, and breast cancer studies [25,26,27]. It is also known to have anti-oxidant, anti-inflammatory and anti-diabetic, antibacterial, antifungal, antitussive, and neuroprotective effects [28,29,30,31,32]. Although TQ is being used for centuries and has been observed to be effective against many disease conditions but its mode of action or molecular target is not known. Previous studies suggested that the dietary benefits of several polyphenolic compounds could be associated with their interaction with ATP synthase. For this purpose, we studied the inhibitory effects of thymoquinone on F_1_F_o_ ATP synthase and the growth of *E*. *coli* cells. Our results show that thymoquinone strongly inhibits ATPase activity and bacterial growth, thereby suggesting that the beneficial effect of thymoquinone as antitumor or antimicrobial agent may in part be linked to its inhibition of ATP synthase.

## Materials and Methods

### Thymoquinone

Thymoquinone with 99% purity (274666-5G) was purchased from Sigma-Aldrich Chemical Company. TQ is unstable in aqueous solution and is light sensitive therefore it was dissolved in DMSO and kept in dark [33]. In ATPase assays the maximal volume of DMSO used was 3.64%. In this study and earlier we noted that up to 40% DMSO by itself has no effect on membrane bound F_1_F_o_ of *E*. *coli* ATP synthase [34]. All other chemicals used in this study were ultra-pure analytical grade purchased either from Sigma—Aldrich Chemical Company or Fisher Scientific Company.

### Growth in limiting glucose medium; preparation of *E*. *coli* F_1_F_o_ membranes; purification of *E*. *coli* F_1_; assay of ATPase activity of membranes or purified F_1_

Purified F_1_ or membrane bound F_1_F_o_ was isolated from the wild-type pBWU13.4/DK8 *E*. *coli* strain [35]. Growth yield on limiting glucose (fermentable carbon source, 3–5 mM) and succinate (non-fermentable carbon source) measuring oxidative phosphorylation was measured as in [36]. In this procedure both wild-type with ATPase gene and null strain (pUC118) in absence of ATPase gene are grown on limiting glucose and succinate. Growth on succinate require ATP synthase so in absence ATPase gene null strain is expected to grow between 40–50% which is due to glycolytic pathway.

**Fig 2:**
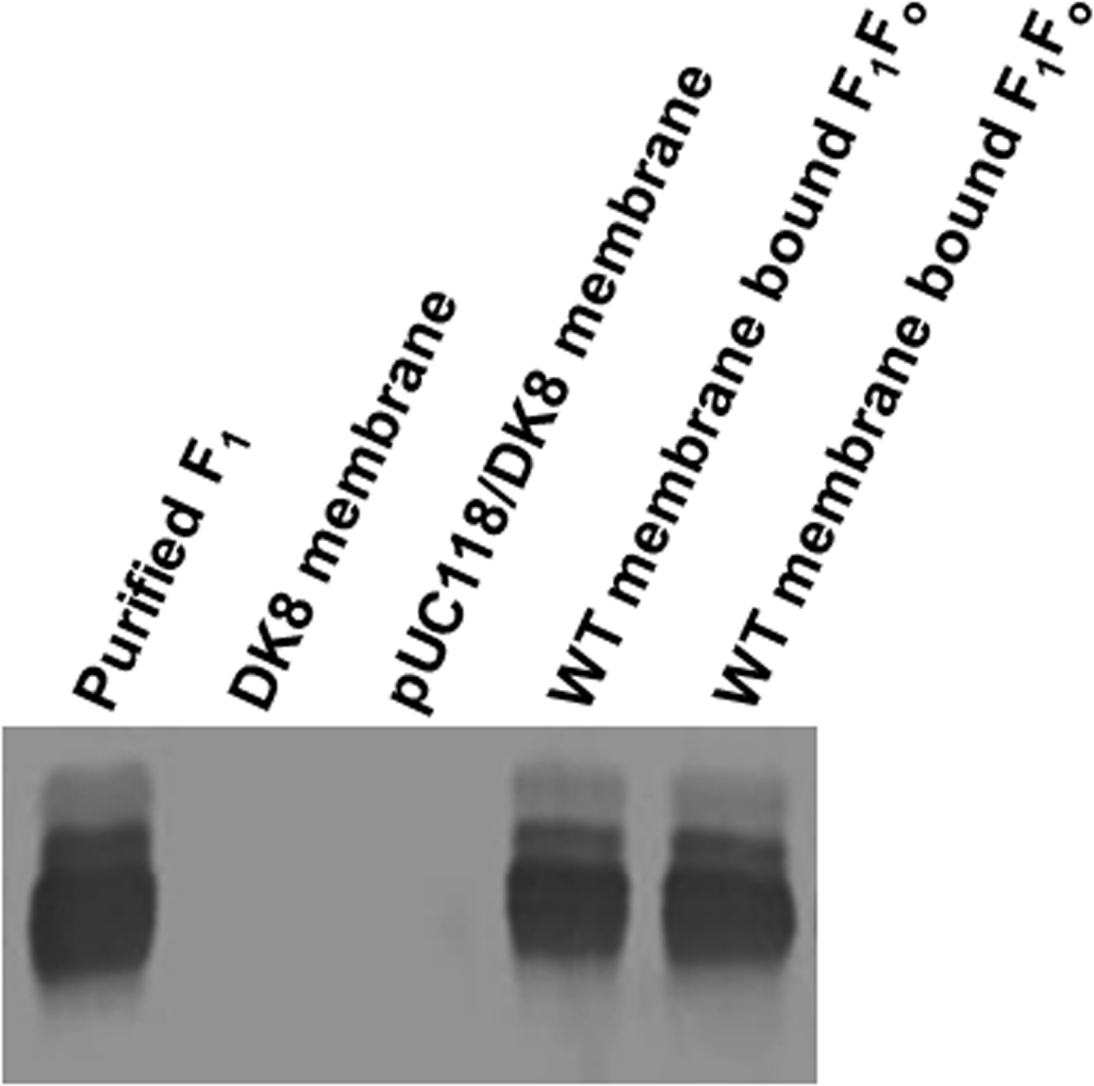
Immunoblotting of wild-type purified F_1_ and membrane bound F_1_F_o_ ATP synthase with anti-F_1_-α antibody. Wild-type purified F_**1**_ (0.4μg) and two membrane bound F_**1**_F_**o**_ preparations (4μg) were run on 10% SDS-polyacrylamide gel with membranes from null mutants DK8 and pUC118/DK8 controls. Protein bands were transferred to nitrocellulose and immunoblotted using anti-F_**1**_-α antibody.

F_1_ F_o_ bound *E*. *coli* membranes were prepared as in [37,38]. This procedure involves three washes of the initial membrane pellets. Wash one in a buffer containing 50 mM TES pH 7.0, 15% glycerol, 40 mM 6-aminohexanoic acid, 5 mM p-aminobenzamidine is followed by two subsequent washes in the buffer containing 5 mM TES pH 7.0, 15% glycerol, 40 mM 6-aminohexanoic acid, 5 mM p-aminobenzamidine, 0.5 mM DTT, 0.5 mM EDTA. Membranes were washed twice more by resuspension and ultracentrifugation in 50 mM TrisSO_4_ pH 8.0, 2.5 mM MgSO_4_ before the experiments. F_1_ was purified as in [39]. F_1_ samples (100μl) were passed twice through 1-ml centrifuge columns (Sephadex G-50) equilibrated in 50mM TrisSO_4_ pH 8.0 to remove catalytic site bound-nucleotide. ATPase activity was measured in 1 ml ATPase cocktail containing 10 mM NaATP, 4 mM MgCl_2_, 50 mM TrisSO_4_, with pH 8.5 at 37°C. Reactions were initiated by the addition of 1 ml ATPase cocktail to purified F_1_ or membranes and stopped by the addition of SDS to 3.3% final concentration. Liberated Pi was measured as in [40]. For membranes (30–50 μg protein), reaction times were 20–30 min. For purified F_1_ (20 μg protein), reaction time was 5–10 min. All reactions were found to be linear with respect to time and protein concentration. SDS-gel electrophoresis (10% acrylamide) and immunoblotting with rabbit polyclonal anti-F_1_-α and anti-F_1_-β antibodies was used to check the integrity and purity of protein (Fig 2) [41,42,43].

### Thymoquinone induced inhibition of ATPase activity

Membranes or purified F_1_ (0.2–1.0 mg/ml) were preincubated with varied concentrations of thymoquinone for 1hour at room temperature, in 50 mM TrisSO_4_, pH 8.0 buffer. The volume of TQ added was in the range of 0–20μl in a total reaction volume of 550μl. Then 1 ml ATPase cocktail was added to measure the ATPase activity. Inhibitory exponential decay curves were generated using SigmaPlot 10.0. The best fit line and IC_50_ value for the curve was obtained using a single 3 parameter model. Statistical significance of the relationship between TQ concentration and enzyme activity was analyzed by linear regression. The range of absolute specific activity for membrane bound F_1_F_o_ was 13–20 and for purified F_1_ was 18–28 μmol/min/mg at 30°C for different preparations. These absolute values were used as 100% bench mark to calculate the relative ATPase activity.

### Reversal from thymoquinone induced inhibition of ATPase activity

Reversibility was measured by dilution of the membranes and by passing the inhibited purified F_1_ through 1ml centrifuge columns. In reversibility by dilution membranes were reacted with 150 μM concentration of thymoquinone for 60 min at room temperature. Then 50 mM TrisSO_4_ pH 8.0 buffer was added to reduce thymoquinone concentration to non-inhibitory levels and incubation continued for an additional 60 min at room temperature before ATPase assay. For purified F_1,_ TQ inhibited enzyme was twice passed through 1 ml centrifuge columns before measuring the ATPase activity. Control samples without TQ were incubated for the same time periods as the samples with TQ.

## Results

### Strong inhibition of *E*. *coli* membrane bound F_1_F_o_ or purified F_1_ ATPase activity by TQ

**Fig 3:**
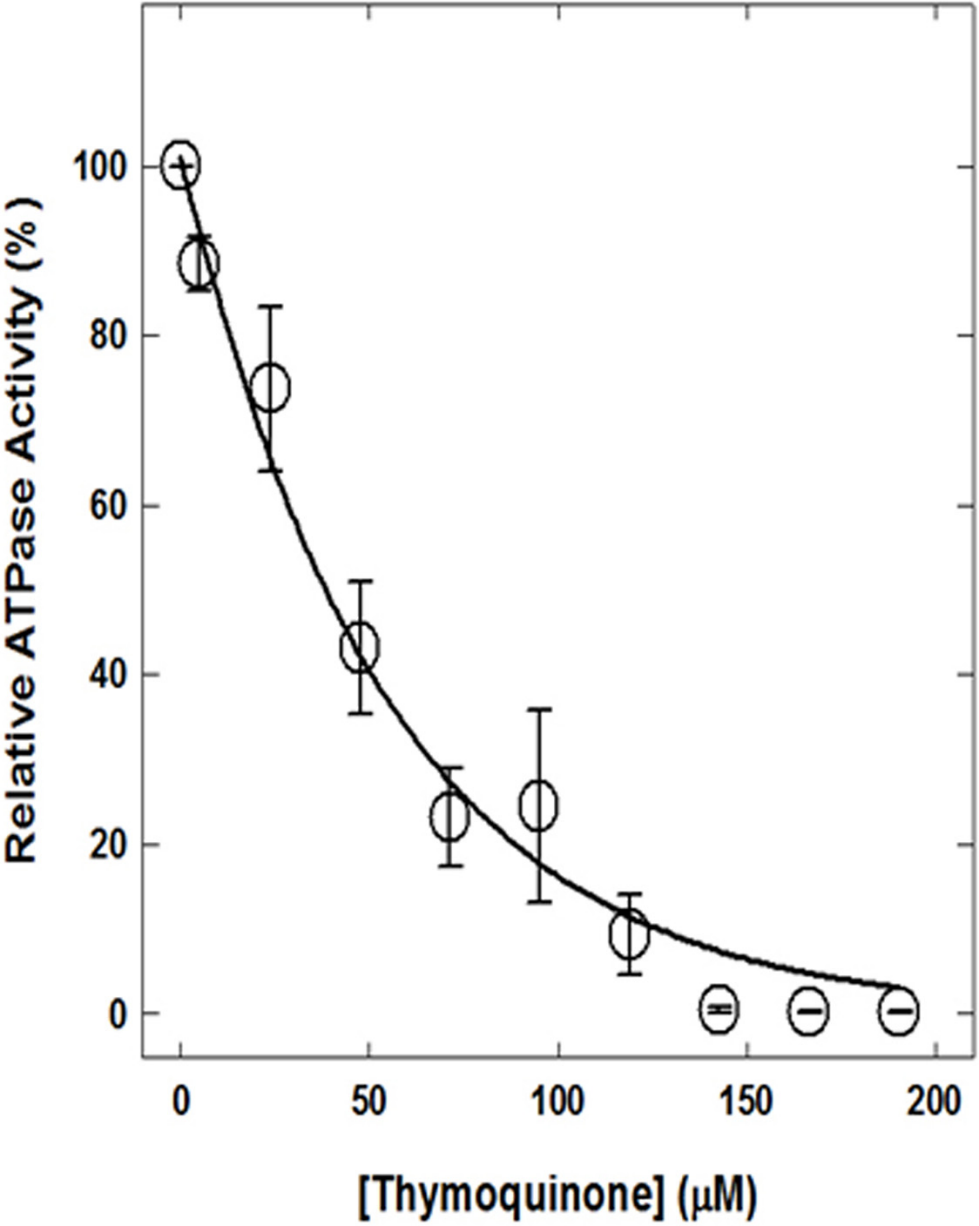
Complete inhibition of ATPase activity of membrane-bound ATP synthase by TQ. Membranes were preincubated for 60 min at 23°C with varied concentration of TQ and then 1 ml of ATPase cocktail was added and activity measured. For details are given in Materials and Methods section. Each data point represents average of four experiments done in duplicate tubes, using two independent membrane F_**1**_F_**o**_ preparations. Thus, mean given with standard error for each inhibitory concentration is N4 where N represents the sample size.

Previously several phytochemicals were shown to bind and inhibit *E*. *coli* ATP synthase [18,19,24,34]. Recently there has been increased interest in TQ regarding its possible therapeutic utility for multiple diseases, particularly as an anticancer or antimicrobial agent. For this reason we studied TQ induced inhibition of ATP synthase. TQ caused complete inhibition of purified F_1_ or membrane bound F_1_F_o_ ATP synthase with ~0.3% residual activity (Fig 3). As shown in Fig 3 there is a significant inverse relationship between TQ concentration and enzyme activity (r = 0.9355; P<0.0001). Maximal inhibition of 99.70% was observed at 150 μM concentration. Each data point represents an average of four experiments, using two independent membrane preparations. The standard error for mean inhibition at varied TQ concentrations did not overlap for virtually all estimates. The maximal standard error of estimates at 95 μM TQ is ±10.5148.

### Reversal of ATPase activity of purified F_1_ or membrane enzyme from thymoquinone inhibition

**Fig 4:**
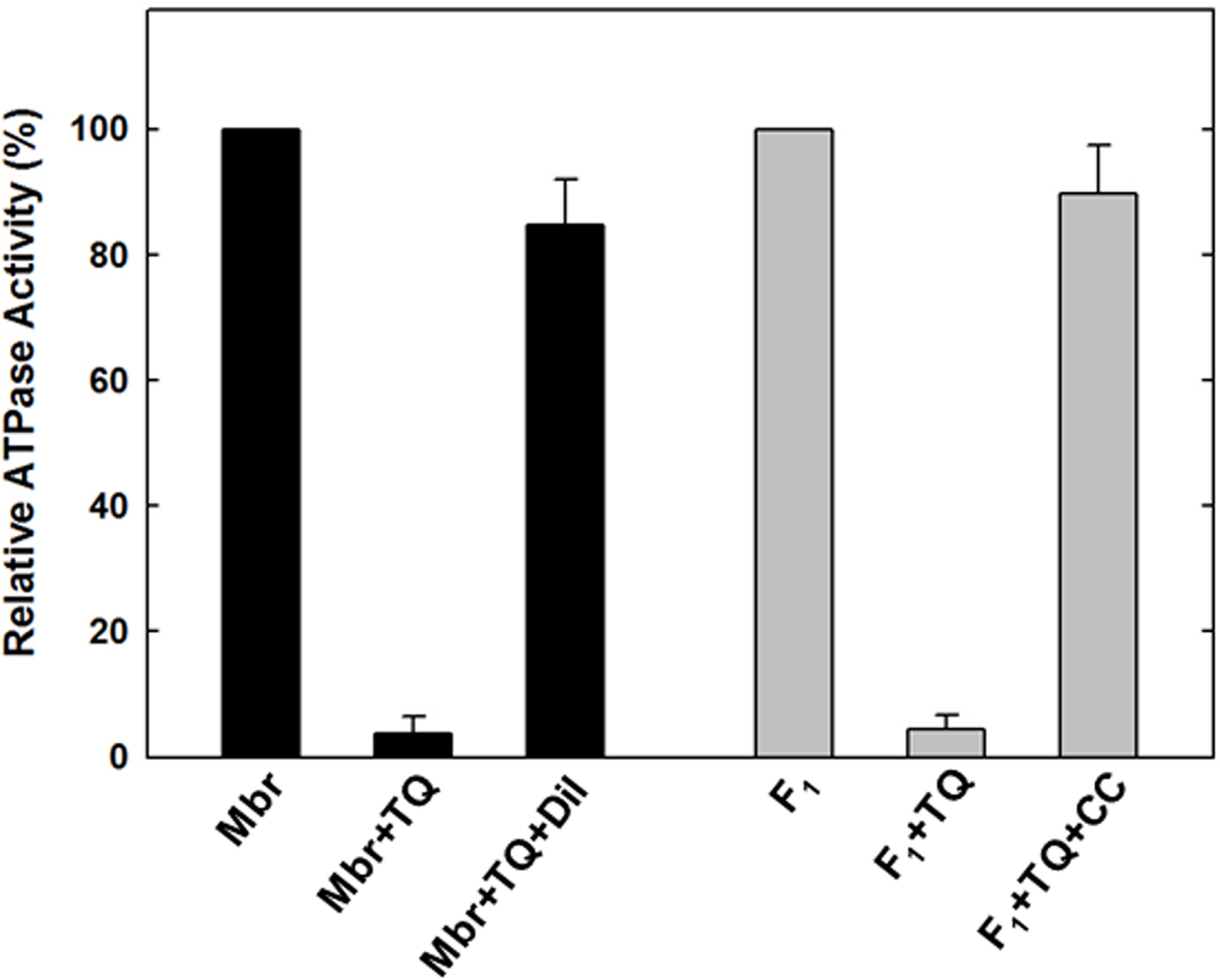
Reversal of TQ induced inhibition by dilution and passing through centrifuge columns. Membrane bound ATP synthase (Mbr) or purified F_**1**_ (F_**1**_) was inhibited with inhibitory concentration of TQ shown in the figure for 60 min under conditions as described in Fig 2. (A), TrisSO4 pH 8.0 buffer was added to bring back the TQ concentration to non-inhibitory level and activity was measured. (B) Purified F_**1**_ was incubated with inhibitory concentrations of TQ for 60 min under conditions as described in Fig 3. Then the inhibited samples were passed twice through 1 ml centrifuge columns and ATPase activity was measured.

TQ induced inhibition of ATP synthase was found to be reversible. Both purified F_1_ or membranes regained activity after dilution of TQ or removal by passing through centrifuge columns (Fig 4). Again the inhibitory concentrations were determined based on data from Fig 3. The inhibited samples were passed twice through 1 ml centrifuge columns and ATPase activity was measured. It was found that activity was restored to the near normal level as in absence of the TQ (Fig 4). Reversibility data indicates that the observed inhibition is not the result of protein denaturation and that the enzyme retains the ability to reactivate upon release of the compound by dilution or removal through centrifuge columns. Such results indicate non-covalent interaction between TQ and ATP synthase.

As shown in Fig 3 for membrane bound F_1_F_o_ ATP synthase the mean maximum inhibition achieved at 150 μM TQ was 96.2% with standard error ± 2.65, while the mean maximum reversal was 84.67 with standard error ± 7.31. For purified F_1_ the mean maximum inhibition achieved at 150 μM TQ was 95.67% with standard error ± 2.33, while the mean maximum reversal was 89.67 with standard error ± 7.86.

### Inhibition of growth on limiting glucose and succinate medium in presence of TQ

As shown in Table 1 TQ potently inhibited the growth of wild-type *E*. *coli* pBWU13.4/DK8 strain in limiting glucose (containing Ile and Val) and succinate (non-fermentable carbon source). 45 to 48% reduction in wild-type growth was observed in presence of 150 μM TQ. No growth inhibition of null strain (pUC118/DK8) by TQ was observed as this strain lacks ATP synthase. Limiting glucose assay contained 3mM glucose and OD_595_ was measured till no further growth occurred which takes about 20 hr time. Growth on succinate plate may take up to 72 hr.

**Table 1:** Thymoquinone (TQ) induced growth inhibition of *Escherichia coli* cells at 150 μM concentration.

Presence/ absence of TQ	^a^Growth on limiting glucose (%)	^b^Growth on succinate (%)	F_1_-ATPase residual activity (%)
^C^Wild-type	100	100	100
^d^Null	44±8	4±3	N/A
Wild-type +TQ	55±10	52±9	0
Null + TQ	45±6	6±4	N/A

^a^Growth yield on limiting glucose was measured as OD_595_ after ~20 hours growth at 37°C.

^b^ Growth on succinate medium after 72 hours was determined by OD_595_

^c,d^Wild-type (pBWU13.4/DK8) contains UNC^+^ gene encoding ATP synthase

^d^Null, (pUC118/DK8) is UNC^-^.

All experiments were done at least three times at 37°C. Individual experimental points are average of duplicate assays.

## Discussion

There is growing interest in the use of natural compounds as antimicrobial and antitumor agents individually or in combination with other such molecules [21,44,45]. Several phytochemicals have been shown to have dietary benefits and are potential anti-tumor or antimicrobial agents [28,31,46]. For centuries TQ has been used as a natural therapeutic product [47,48]. The goal of this study was to determine if the antimicrobial or anticancer properties of TQ may be associated with the inhibition of ATP synthase. Therefore, we examined TQ effects on ATPase activity and on growth inhibition profiles of *E*. *coli* to examine the potential of ATP synthase as a molecular target.

TQ fully inhibited both purified F_1_ and membrane bound F_1_F_o_ ATP synthase with IC_50_ ~36.95μM (Fig 3). This is in agreement with multiple previous studies where it was shown that the inhibitory profiles of both F_1_F_o_ membrane preparations as well as isolated purified F_1_ are the same [24,49,50,51,52,53]. It is interesting to note that in a previous study simple phenolic compounds, dihydrothymoquinone, hydroquinone, resorcinol, or catechol, structurally related to TQ, resulted in partial or incomplete inhibition of ATP synthase [54]. Resveratrol, piceatannol, and quercetin inhibited ATP synthase X-ray crystal structures show that the polyphenol binding pocket for resveratrol, piceatannol, and quercetin is contributed by residues from α, β, and γ-subunits [19]. Moreover, several polyphenolic compounds structurally related to TQ (Fig 1) were previously shown to bind to the polyphenol binding pocket [24,34,54] identified by Walker and colleagues [19]. Therefore, there is a high possibility that the-CH_3_ group of TQ forms hydrophobic non-polar interactions with γGln274, γThr-277, βAla-264, βVal-265, γAla-270, γThr-273, γGlu-278, αGly-282, or αGlu-284 residues. TQ bound X-ray structure of ATP synthase and or mutagenic analysis of above residue should be able to confirm the involvement of above residues in TQ binding. TQ induced inhibition was also found to be completely reversible. Passage through centrifuge columns dissociates TQ from the inhibited F_1_ and resulted in restored enzyme function. Dilution of purified F_1_ or membrane lowers inhibitor concentration and allowed recovery of ATPase activity. These results indicate that the interaction between inhibitor and the enzyme is non-covalent, as has been observed in previous studies examining the inhibition of ATP synthase by several polyphenolic molecules [24,34,54].

Black seeds (*Nigella sativa*) have been used for centuries in traditional medicine to treat many disease conditions, including bronchial asthma, dysentery, infections, and hypertension [47]. So far a number of components from black seed such as thymohydroquinone, dithymoquinone, thymol, and TQ have been isolated and characterized. TQ has been shown to have antioxidant, anti-inflammatory, and chemopreventive properties [27,28,55]. As an anticancer agent TQ extracted from black seed was shown to act against lung, breast, and melanoma cancer cells [27,28]. It was also shown that TQ potently inhibited pathogenic and nonpathogenic bacterial growth and was suggested that TQ inhibits biofilm formation. However, the mechanism by which TQ affects biofilm formation is not known [31,56]. It is quite possible that biofilm production is affected through the inhibition of the F_o_ part of ATP synthase, as was the case with *Streptococcus mutans*, where inhibition of ATP synthase of *S*. *mutans* inhibited biofilm formation and acid production [23]. Also, TQ was shown to have very selective antimicrobial activity and showed about a four-fold enhanced synergistic effect in combination with other antibiotic drugs against oral pathogens [30]. TQ was found to inhibit the migration of human and mouse metastatic melanoma cells [46]. TQ was also shown to have a role in decreasing hepatic gluconeogenesis and in normalization of the dysregulated insulin production observed in HAART treated patients [29,57].

TQ induced growth inhibition of *E*. *coli* cells corroborated the F_1_-ATPase inhibition by TQ (Table 1). Null strain (pUC118/DK8) typically shows 40–50% growth in comparison wild-type, (pBWU13.4/DK8). Null strain growth uses glycolysis to generate ATP, whereas the wild-type grew using glycolysis, TCA, and oxidative phosphorylation. TQ reduced wild-type growth between 45 to 48% in limiting glucose and succinate media respectively, but had nearly no effect on the null strain. Growth retention in both wild-type and null cells can be attributed to ATP production through the glycolytic pathway. Moreover, loss of growth in wild-type results from loss of oxidative phosphorylation through inhibition of ATP synthesis by TQ. Growth inhibition of wild-type in succinate as the sole carbon source in the presence of TQ supported the inhibition of F_1_-ATPase activity. These results demonstrate that TQ induced inhibition of microbial growth is through the inhibition of ATP synthase.

Our results suggest that dietary benefits of TQ in part may be linked to its inhibitory effects on ATP synthase. Inhibition of bacterial cell growth in the presence of phytochemicals like bioflavonoids [18,24,34], and TQ from this study suggests ATP synthase as a potential drug target for dietary bioflavonoids and TQ. TQ has been reported to be effective in multiple disease conditions, suggesting TQ as a potential therapeutic molecule for those diseases. Mode of action though is not clear in many cases. Based on abrogation of ATPase activity and growth inhibition assays we conclude that the dietary benefits of TQ may be related at least in part to its action through the binding and inhibition of ATP synthase.
